# Management of non-gonococcal urethritis

**DOI:** 10.1186/s12879-015-1043-4

**Published:** 2015-07-29

**Authors:** Harald Moi, Karla Blee, Patrick J Horner

**Affiliations:** Olafia Clinic, Oslo University Hospital, Institute of Medicine, University of Oslo, Oslo, Norway; Bristol Sexual Health Centre, University Hospitals Bristol NHS Foundation Trust, Bristol, UK; School of Social and Community Medicine, University of Bristol, Bristol, UK

**Keywords:** Non-gonococcal urethritis, Mycoplasma genitalium, Chlamydia trachomatis, Urethral smear microscopy, Macrolide resistance

## Abstract

Non-gonococcal urethritis (NGU), or inflammation of the urethra, is the most common treatable sexually transmitted syndrome in men, with approximately 20-50 % of cases being due to infection with *Chlamydia trachomatis* and 10-30 % *Mycoplasma genitalium*. Other causes are *Ureaplasma urealyticum*, *Trichomonas vaginalis*, anaerobes, Herpes simplex virus (HSV) and adenovirus. Up to half of the cases are non-specific. Urethritis is characterized by discharge, dysuria and/or urethral discomfort but may be asymptomatic. The diagnosis of urethritis is confirmed by demonstrating an excess of polymorpho-nuclear leucocytes (PMNLs) in a stained smear. An excess of mononuclear leucocytes in the smear indicates a viral etiology. In patients presenting with symptoms of urethritis, the diagnosis should be confirmed by microscopy of a stained smear, ruling out gonorrhea. Nucleid acid amplifications tests (NAAT) for *Neisseria gonorrhoeae, C. trachomatis* and for *M. genitalium.* If viral or protozoan aetiology is suspected, NAAT for HSV, adenovirus and *T. vaginalis,* if available. If marked symptoms and urethritis is confirmed, syndromic treatment should be given at the first appointment without waiting for the laboratory results. Treatment options are doxycycline 100 mg x 2 for one week or azithromycin 1 gram single dose or 1,5 gram distributed in five days. However, azithromycin as first line treatment without test of cure for *M. genitalium* and subsequent Moxifloxacin treatment of macrolide resistant strains will select and increase the macrolide resistant strains in the population. If positive for *M. genitalium,* test of cure samples should be collected no earlier than three weeks after start of treatment. If positive in test of cure, moxifloxacin 400 mg 7–14 days is indicated. Current partner(s) should be tested and treated with the same regimen. They should abstain from intercourse until both have completed treatment. Persistent or recurrent NGU must be confirmed with microscopy. Reinfection and compliance must be considered. Evidence for the following recommendations is limited, and is based on clinical experience and guidelines. If doxycycline was given as first therapy, azithromycin five days plus metronidazole 4–500 mg twice daily for 5–7 days should be given. If azithromycin was prescribed as first therapy, doxycycline 100 mg x 2 for one week plus metronidazole, or moxifloxacin 400 mg orally once daily for 7–14 days should be given.

Urethritis, or urethral inflammation, is in the majority of cases caused by sexually transmitted agents. Typical symptoms are discharge, dysuria, urethral stinging/itching and penile tip irritation, but urethritis is often asymptomatic [1].

The diagnosis should be confirmed with microscopy of a stained smear from urethra, demonstrating an excess of polymorphonuclear leucocytes (PMNLs) or monocytes in the anterior urethra [1]. Urethritis may be gonococcal, when *Neisseria gonorrhoeae* is detected or non-gonococcal (NGU). Mucopurulent non-gonococcal cervicitis and urethritis is the female equivalent [2–4]. However, the definition of cervicitis and female urethritis is controversial [4].

## Aetiology

*Chlamydia trachomatis* is, in most settings, the only aetiological bacterial agent causing urethritis which is routinely tested for, and isolated in 20–50 % [5]. *Mycoplasma genitalium* has emerged as the second most common aetiological agent, causing 10–30 % of cases [5–7], with a double infection with *C. trachomatis* in 5 to 15 % [8]. In 30–60 % of the cases with NGU neither *C. trachomatis* nor *M. genitalium* is detected [9, 10]. Pathogen negative NGU is more likely with increasing age, the absence of discharge or clinical symptoms, and engagement in low-risk practices [9–11].

In Western Europe, *Trichomonas vaginalis* as a cause of male urethritis is uncommon [12]. In the United States, *T. vaginalis* is associated with Afro-American race, with prevalences of 2.5–17 [11, 13, 14]. Ureaplasmas are inconsistently associated with NGU [15]. Earlier studies did not differentiate between the two species *Ureaplasma urealyticum* (biovar 2) and *U. parvum* (biovar 1). There is increasing evidence that *U. parvum* is non-pathogenic, and only *U. urealyticum* is pathogenic in some men but not all men colonized in the urethra [16–18]. The immune response may influence the development of NGU as in one study an association of *U. urealyticum* with NGU was only observed in men with fewer lifetime sex partners compared to those who had many [17]. Inoculation with *U. urealyticum* has been shown to give urethritis symptoms [19]. Higher organism load (>1000 copies/ml of FVU) of *U. urealyticum* may be a predictor of NGU [20]. *U. urealyticum* may account for 5–10 % of cases of acute NGU, but is often detected without urethritis, and therefore screening and test of cure for this micro-organism is questionnable [21]. Adenoviruses may account for 2–4 % of symptomatic patients and this is often associated with conjunctivitis [22–24]. Herpes simplex viruses types 1 and 2 are uncommon causes of NGU (2–3 %) in the absence of typical genital ulceration [24]. If aetiology of the urethritis is viral, monocytes are present in the microscopical smear [23]. *N. meningitidis*, *Haemophilus sp.*, *Candida sp*., urethral stricture and foreign bodies have all been reported in a few cases and probably account for a small proportion of NGU, whilst the role of Epstein Barr Virus is questionable [25, 26]. There is emerging evidence that bacterial vaginosis-associated bacteria may be associated with NGU [27]. It is unclear what causes organism-negative NGU or idiopathic urethritis. Some idiopathic cases are almost certainly non-infective, but we do not currently have the tools to be able to differentiate probably non-infective from likely infective cases [28]. With modern techniques a diverse microbiome can be detected in the male urethra, and there may still be unknown bacteria causing urethritis [28, 29].

## Clinical features and signs

Symptomatic patients and, if observed, those with a visible discharge should be assessed for the presence of urethritis. The discharge is mucopurulent and often sparse, but may be cloudy or clear. An excessive purulent discharge should arouse suspicion of gonorrhoea.

Formally, the definition of urethritis diagnosis is microscopically, demonstrating ≥5 PMNL per high power (x1000) microscopic field (averaged over five fields with greatest concentration of PMNLs) [30] in a stained smear from the anterior urethra. This should be performed in the office as guidance for immediate treatment, without waiting for the laboratory results.

Gram stain is used in most countries. Methylene blue stain, which is much more rapid and easier to perform in the examination room, is used in the Nordic countries [6]. Differentiating gonococcal urethritis from NGU has been shown to be equal with the two staining methods [31].

The quality of the smear is heavily dependent on how the smear is taken and there is both inter- and intra-observer variation when interpreting the result [32, 33]. A 1–5 mm plastic loop, a sterile blunt spatula or cotton or rayon tipped swab can be used. The smear should be taken about 0.5 to 1 cm into the urethra. A plastic loop is less painful than a Dacron swab, which is less painful than a Rayon swab [34]. Based on reported sensitivities of microscopy for detection of CT, blunt spatula is better than swab [35, 36]. If a urethral discharge is present and can be adequately sampled without placing the loop, spatula or swab inside the meatus, this would be the recommended method for preparing a smear as it is likely to be preferred by the patient [37]. However, this has not been compared to the standard technique in a clinical trial.

Traditionally, a cut-off of 5 PMNL/high power field microscopy (HPF) has been used for the diagnosis of urethritis, but this has recently been questioned. A high prevalence of *Chlamydia trachomatis* has been observed in cases of low-grade urethritis with 3–5 PMNL/HPF [36, 37]. However, a possible reason for the low sensitivity in these studies may be the sampling methods, using a swab or un-identified device instead of a spatula [6, 24, 35–37].

## Investigations

All cases of urethritis should be screened for *C. trachomatis* by NAAT on first void urine. *N. gonorrhoeae* must be ruled out with urethral smear microscopy, NAAT and culture. In addition, if available, specific test for *M. genitalium* should be taken, preferably including test for macrolide resistance of *M. genitalium* [38]. If suspected because of clinical or epidemiological reasons, tests for *Trichomonas vaginalis,* adenovirus and HSV should also be included.

Men who have sex with men (MSM) should be tested for *C. trachomatis* and *N. gonorrhoeae* from any potentially exposed site [39]. All tests positive for *C. trachomatis* from the rectum in MSM should, if possible, be subtyped for lympho-granuloma venereum.

The first void urine should not contain more than 10 ml, a higher volume will decrease the sensitivity. For chlamydia the time after micturition do not seem to matter, but for *M. genitalium* two hours is conventional.

A urinary tract infection (UTI) is rare in young males, and screening for a UTI in young men with urethritis is not indicated. If the patient complains of severe dysuria, haematuria (microscopic or macroscopic), nocturia, urinary frequency, urgency, or is at low risk for a sexually transmitted infection, a urinary dipstick analysis on a mid-stream urine specimen should be considered and a mid-stream urine sent for culture and sensitivities [40]. If a UTI is found, young men should be investigated for urinary tract abnormalities. Use of a leucocyte esterase dipstick on the remains of the FVU specimen is not recommended for diagnosis of NGU, since it does not have adequate sensitivity to be considered a reliable rapid diagnostic test [41]. Symptomatic patients negative in routine NAAT should be referred to a centre which has microscopy available.

## Symptomatic patients with a normal urethral smear

Treatment without etiological diagnosis or verifying the presence of urethritis is not recommended. The sensitivity of the smear test for diagnosing urethritis is affected by the period since last passing urine. The optimum time to ensure a definite diagnosis in a symptomatic man is not known, 2–4 hours is conventional. If urethral smear is normal and all specific tests are negative, the patient can be advised to re-attend for an early morning smear (EMS) if his symptoms do not settle. He should hold his urine overnight and attend not having voided urine. It is good practice to advise the patient to take their last drink about 8 pm and to void about 3 hours later in order to help avoid waking with a full bladder.

## Complications

In men younger than 40 years-of-age with acute epididymitis, *C. trachomatis* is the major non-gonococcal pathogen. The role of genital mycoplasmas and ureaplasmas in the development of acute epididymitis remains to be determined [42].

Sexually acquired reactive arthritis is infrequent and depends on the prevalence of HLA B27 in the population [43].

## Treatment

The most important step in syndromic management of urethritis is determining whether *N. gonorrhoeae* is present during the initial clinic visit. Despite some concerns about sensitivity, stained urethral smears for microscopic identification of *N. gonorrhoeae* is recommended for concurrently documenting inflammation and the presence of intracellular diplococci [31]. Subjects without signs of *N. gonorrhoeae* infection are managed syndromically. In men with severe symptoms the treatment should be initiated as soon as the diagnosis is made, without waiting for the laboratory test results. In men with mild symptoms, a potential option is to review the patient after 3–7 days, when the results of the NAAT(s) are available, as sometimes urethritis can resolve without treatment [44]. If laboratory tests are positive, or in the case of persistent microscopic urethritis, appropriate antimicrobial treatment, depending on which microorganism is detected, can then be administered at the second visit [45]. If the laboratory tests are negative, only treat if patient has symptoms and microscopic evidence of urethritis, or observable purulent or muco-purulent discharge on examination.

Ideally, treatment should be effective (microbiological cure >95 %), easy to take (not more than twice daily), with a low side effect profile, and minimal interference with daily lifestyle. However, assessing treatment efficacy is not straightforward. Detectable inflammation may persist for an unknown length of time without persistent infection, and persistent infection may not result in persistent NGU [15, 46–48]. Two recent randomised controlled trials from the United States observed that the clinical response rate of doxycycline 100 mg x 2 for one week and azithromycin 1 gram stat was the same [49, 50]. *Mycoplasma genitalium* treatment failure was extremely common. A meta-analysis of chlamydia treatment options showed a slightly better cure rate of 3 % with doxycycline compared with single dose azithromycin for uncomplicated chlamydia infection, but a 7 % better cure rate with doxycycline for clinical urethritis [51].

There are two regimens for azithromycin treatment, single one gram dose, or a five days course – 500 mg day one followed by 250 mg day 2–5. Single dose azithromycin is thought to induce macrolide resistance in *M. genitalium* to a higher extent than the five days treatment [52], and should be avoided if *M. genitalium* is not ruled out. An initial dosage of one gram azithromycin followed by 250 mg day 2–5 has also been suggested. However, there are no randomized studies comparing the different regimens in men with acute NGU so the recommendation to use a 5 day regimen with 1 gram initial dosage is based on expert opinion alone.

Doxycycline has a somewhat higher cure rate in *C. trachomatis* than azithromycin [49, 50], cures 20–40 % of *M. genitalium* without inducing macrolide resistance, and should be used as first line treatment.

Azithromycin is widely used as first line treatment for NGU, but seems to have contributed to the increasing *M. genitalium* resistance*.* Azithromycin, especially the single 1 gram regimen, will induce macrolide resistance in some *M. genitalium* strains [52], and will cure only the macrolide susceptible strains. Thus, without a test of cure (TOC) for *M. genitalium* and subsequent moxifloxacin treatment of treatment failures, the macrolide resistant strains will be selected and further spread. As a consequence, the proportion of macrolide resistant strains in the population will increase. The extensive use of azithromycin 1 gram for treatment of NGU without a TOC for *M. genitalium* may explain the declining cure rates [49]. In Greenland, with a small population of 55,000 inhabitants, a very high incidence of NGU and extensive use of single dose azithromycin, the macrolide resistance rate of *M. genitalium* has been shown to be 100 % [53], with a high and comparable incidence of chlamydia and *M. genitalium.*

Lymecycline or tetracycline hydrochlorine do not induce photosensitivity and can be prescribed if sun exposure cannot be avoided [54].

## Sexual contacts/partners

All sexual partners at risk should be assessed and offered epidemiological treatment, maintaining patient confidentiality. The duration of “look back” is arbitrary; 4 weeks is suggested for symptomatic men. However, 12 months has been suggested for chlamydia [55]. Partner(s) notification and management should be carried out with sensitivity, considering socio-cultural issues and avoiding stigma.

Current partner(s) should be tested and treated and the patient advised not to be sexually active until one week after both have initiated treatment.

If *C. trachomatis, M. genitalium* or *N. gonorrhoeae* are detected, it is important to ensure that all sexual partner(s) potentially at risk have been notified.

## Follow up for patients with NGU

All patients should be followed up to ensure completion of partner notification and ensure they are free of symptoms. This follow up can be done by telephone.

Test of cure samples should be collected no earlier than three weeks after start of treatment in those who tested positive for *M genitalium* [49, 56–58]. A repeat test for chlamydia may be recommended after 3–12 months in order to detect reinfections. Patients who remain symptomatic should be asked to return to the clinic and retreated with appropriate regimen (see below) and the possibility of re-infection explored.

## Management and diagnosis of recurrent NGU

Ensure that the patient has completed the initial course of therapy and that reinfection is not a possible cause. Only treat if patient has symptoms and microscopic evidence of urethritis, or observable purulent or muco-purulent discharge on examination. Reassure asymptomatic patients that no further test or treatment is necessary.

Gram or methylene blue stained urethral smear should be taken only in men who are symptomatic. If symptomatic and normal microscopy an EMS is recommended. Consider testing for *Trichomonas vaginalis* using a NAAT if available, and testing for *M. genitalium* including screening for macrolide resistance if identified. Symptomatic patients negative on routine NAAT without an obvious discharge should not be retreated without microscopic confirmation, and should be referred to a centre which has microscopy available.

## Persistent NGU

The aetiology of persistent NGU is probably multifactorial with an infectious agent being identified in <50 % of cases [11]. *M. genitalium* has been identified in 20–40 % [6–8], [11] and *C. trachomatis* in 10–20 % in men treated with azithromycin 1 g [49]. *U. urealyticum* may also play a role in some men [15–17]. *Trichomonas vaginalis* can be identified in up to 10 % in populations where it is endemic, but is rare in Western Europe [12]. Recurrent herpes simplex virus infection should be considered, as this can cause dysuria without signs outside the urethra [24].

Any treatment of persistent NGU should cover *M. genitalium, T. vaginalis* and anaerobes*,* if not tested and treated for at initial presentation.

As there is no evidence that female partners of men with persistent/recurrent NGU are at increased risk of pelvic inflammatory disease, the historical advice has been that they do not need to be retreated if treated appropriately at first. However, in view of the emerging evidence that persistence of *M. genitalium* post azithromycin may occur in 20 to 40 % of men and women and doxycycline is < 50 % effective [49], it is likely that re-treatment of both the sexual partner and index case will be beneficial if persistent/recurrent NGU in the index case resolves following extended therapy but subsequently recurs. For a male partner this would be the same. This remains an area where further research is needed.

## Treatment of persistent or recurrent NGU

If reinfection is unlikely at follow-up, the patient has completed the initial course of therapy, is symptomatic and an observable discharge is present or microscopic evidence of urethritis is confirmed, re-treatment should be given. Any treatment of persistent NGU should cover *M.*

*genitalium* and *T. vaginalis* and/or bacterial vaginosisassociated bacteria. However, evidence for the following recommendations is limited, and is based on clinical experience and guidelines [59, 60]. If doxycycline was prescribed as first line therapy, switch to Azithromycin 500 mg or 1gram day one, then 250 mg once daily for 4 days plus metronidazole 4–500 mg twice daily for 5 days. If azithromycin was prescribed as first line therapy, doxycycline 100 mg twice daily for 7 days plus metronidazole 4–500 mg twice daily for 5–7 days should be prescribed. However, if positive for *M. genitalium* at TOC after azithromycin treatment without suspected re-infection, or in case of positive macrolide resistant test, Moxifloxacin 400 mg orally once daily for 7–14 days should be given.

Moxifloxacin should be used with caution and reserved for treatment failures which are thought secondary to macrolide resistant *M. genitalium*, because of rare but serious adverse hepatic reactions. In patients having contracted *M. genitalium* infection in South-East Asia, dual resistance to both macrolides and quinolones is currently around 10 %. Such infections are difficult to treat and only pristinamycin (registered in France) has proven effective on a case basis [61].

## Continuing symptoms

There is only limited evidence on how best to manage patients who either remain symptomatic following a second course of treatment or who have frequent recurrences after treatment. Reassure asymptomatic patients that no further test or treatment is necessary.

To guide management, testing for *M. genitalium,* including macrolide resistance test, is essential. All sexual partners should be treated concurrently with the same antibiotic regimen which was effective in the index [62].

Recurrent herpes simplex urethritis should be ruled out, especially if the main symptom is dysuria, and many monocytes are seen in the smear [23, 24].

Chronic abacterial prostatitis and the chronic pelvic pain syndrome and psychosexual causes should be considered in the differential diagnosis [62, 63]. Chronic pelvic pain syndrome is a complex condition which overlaps with chronic urethritis and a biopsychosocial, holistic management strategy incorporating evidence-based pharmacotherapy has recently been demonstrated to be effective in managing such patients [64, 65].

For men with persistent or recurrent urethritis, there is currently no evidence that re-treatment of an appropriately treated sexual partner is beneficial (see above). However, it would be prudent to retreat the partner if the man with chronic NGU is cured following extended therapy but subsequently recurs on sexual intercourse. In this scenario the sexual partner, if female, should be examined and tested for *Trichomonas vaginalis* and bacterial vaginosis (BV), regardless of symptoms, and both should be retreated. Doxycycline may be more effective than azithromycin against the newly identified BV-associated bacteria [27].

Urological investigation is usually normal and is not recommended unless the patient has urinary flow problems [62, 63].

## Conclusions

In patients presenting with symptoms of urethritis, the diagnosis should be confirmed by microscopy in order to demonstrate an excess of PMNLs in a stained smear and rule out gonorrhoea. Detection of N. gonorrhoeae, C. trachomatis and for M. genitalium using a NAAT is indicated in all patients. If viral or a protozoan aetiology is suspected, HSV, adenovirus and T. vaginalis should also be tested for using a NAAT, if available. Syndromic treatment should be given on confirmation of the diagnosis in men with marked symptoms.  Treatment options are doxycycline 100 mg x 2 for 7 days or azithromycin 1 gram or, 1.5 grams over 5 days. Although azithromycin 1gram continues to be recommended in national guidelines it is associated with development of M. genitalium macrolide resistant strains and 500mgs then 250mgs od for 4 days (1.5 gram) may be a more sensible option. For men in whom M. genitalium is detected a repeat NAAT test is indicated no earlier than three weeks after start of treatment. For those men with persistent infection,  moxifloxacin 400 mg 7–14 days is indicated. Current partner(s) should be tested and treated with the same treatment regimen given to the index case. They should abstain from intercourse until completion of treatment. Persistent or recurrent NGU must be confirmed with microscopy. Reinfection and compliance should be considered as possible reasons for treatment failure. The following treatment options are based on clinical experience and expert opinion. If doxycycline was given as first line therapy, azithromycin 1.5 grams over five days plus metronidazole 4–500 mg twice daily for 5–7 days should be given. If azithromycin was prescribed as first line therapy, doxycycline 100 mg x 2 for one week plus metronidazole should be given. Moxifloxacin 400 mg orally once daily for 7–14 days can be used if macrolide resistant M. genitalium infection is suspected. 
